# Lecture on Cancer Cures and Cancer Curers

**Published:** 1857-11

**Authors:** T. Spencer Wells


From the Virginia Medical Journal.

*Lecture on Cancer Cures and Cancer Curers.* Delivered at the Grosvenor Place School of Medicine, July 8, 1857. By T. SPENCER WELLS, F. R. C. S.

GENTLEMEN—So much has been said lately about certain pretended cures of cancer, and so much interest has been excited by trials of a variety of caustics, with the hope that they might replace the knife in the removal of cancerous tumors, that I think you should be acquainted with the present state of the question. I shall, therefore, devote this lecture specially to an account of some of the persons who have been most notorious as "cancer curers," making a few remarks on the relative value of the knife and caustics in the treatment of cancer, and conclude by pointing out such means as may enable you in many cases to dispense with either one or the other.

The cases you now see are, I presume, chiefly in hospital practice, where you are not likely to meet with the "cancer curers." I may as well, therefore, give you one or two instances of the sort of cases you may meet with in private. In the year 1853 I was sent for to see a gentleman well known in the higher ranks of London society. He had a malignant tumor beneath the angle of the left jaw, and his case is so excellent an example of the way in which people with cancer run about, first among the surgeons and then to the cancer curers, that I will relate it at some length. This gentleman first complained of a sore in the inside of the left cheek. He fancied he had bitten it. Then it was thought that a decayed tooth had caused it, and the tooth was extracted. Then caustic was used. Still it did not get well, and Mr. Fergusson was consulted. He excised the diseased part; a good deal of bleeding followed, and styptics were used freely. Some time after, swelling came on beneath the jaw, and Mr. Lawrence was consulted. He said the disease was malignant, advised attention to the general health, and a course of sarsaparilla. Then I was called in. The skin at that time was on the point of giving way. I employed congelation by Dr. Arnott's process. This did some temporary good. It gave relief to pain, and I felt pretty sure that it retarded the growth of the tumor. However, the patient was not content with that, and Dr. Marsden was sent for. He advised a very generous diet. Soon after this the skin gave way, and carrot poultices were used. Then came the turn of the cancer curers. Dr. Pattison was sent for, and had sense enough to see that he could do nothing, but made the most of the case, of course, by saying that if he had been called in at first he should certainly have effected a cure. Then a German empiric was heard of who was doing wonders somewhere on the Rhine, and he was written to. He offered to come over for five hundred pounds, and ultimately an arrangement was made to give him three hundred. He came, saw, and conquered—not the disease, but the patient. He applied a very strong caustic one Monday; on Tuesday it had destroyed the coats of a large artery, which gave way, and the patient bled to death in a very few minutes.

Every one who has seen much practice in town could tell such stories as these, but one I have heard Dr. Jenner relate is most striking. He was called one morning, seven or eight years ago, to see a lady who was said to have fainted. He fonnd a lady dead in bed, and a cancer curer just about to re-apply a dressing upon the breast of the dead woman.—This person was so ignorant of medicine that he did not know she was dead; he was horror-struck when Dr. Jenner told him so, and had just before assured the husband that his wife was going on well, and would soon be cured. The quack was not punished. The husband and friends were ashamed of having been duped, and they kept quiet.

Hume is not the first nor the only philosopher who has remarked how constantly mankind is deceived by the very same tricks played over and over again. "In spite of all warning, we see one generation after another, with their eyes wide open, walk into the same gulf of fraud, quackery and imposture." This is especially true in regard to medicine. Large fortunes have been made by the sale of a single nostrum. Perkins sold thousands of his " tractors " at five guineas a pair. Mesmer, and Deslon, his pupil, magnetized many thousands of pounds into their pockets. The fortunes made by Mayersbach and Schweinforth in London in the last century were splendid, and at the present day more than one empiric has the art of attracting crowds from every part of Europe, to some obscure German village. Some of these people may not do much absolute harm, but we are not without instances of victims to quackery in every class of life. Horace Walpole gives us a list of several distinguished sufferers. " Sir Robert Walpole," says Horace, " was killed by a lithotripic medicine; Lord Bolingbroke by a man who pretended to cure him of cancer in the face; and Winnington died some time after, by the ignorance of a quack who physicked and bled him to death in a few days for a slight rheumatism." There is no man now in large practice who could not add to this list some victim of the quackish follies of the day; and it becomes our duty to enquire why the public are so apt to think favorably of those who profess to possess secret remedies for diseases which are considered by regularly educated members of the medical profession to be incurable.

Now there are various reasons for the success of empirics; but the principal reason is, that in many cases where honest men give no hope, the quacks promise health and life; and the patients, like drowning men, catch at every twig and shadow. Then there is a love of novelty, and the benevolent desire to promote any thing which promises to relieve pain or save life; and an English feeling of giving fair play to everybody, and not allowing any class of men to exercise a monopoly of the healing art. Then self-love comes into play. People are apt to be led away by confident assertions; they espouse a scheme warmly, and say so much in its favor, that when the bubble bursts they find it difficult to confess their error. When the imposition is discovered, the partisan is ashamed of having been duped, and holds his tongue. Even those who have suffered both in health and pocket think it better, for the sake of their own reputation as sensible people, to keep quiet. Probably such reserve would be less frequent, if it were considered that others suffer from a perpetuation of the delusion, and the deceptions would be acknowledged as soon as discovered. This duty, however, is overlooked; for while actions for malpraxis are not unfrequent against medical men, it is rare to find an empiric arraigned. Yet the late Lord Gardenstone took the trouble to enquire for a number of persons who had actually attested marvelous cures, and found that more than two-thirds of the number "died very shortly after they had been cured;" and I shall show you presently that we are not without similar results of equally marvelous cures in our own day. I think this should teach us all a lesson. We have chosen the profession of medicine. It is our duty, our business, to study how to cure disease; and we ought never to look upon any disease as incurable, never to give up any case as entirely hopeless; for "the extinction of hope is the extinction of endeavor." Let me impress upon you, then, the duty of looking upon diseases commonly considered incurable in a more hopeful spirit—regarding them only as incurable because our art is imperfect, and of searching diligently for a remedy which may remove the imperfection. We may thus keep hope alive—we may alleviate where we cannot cure, soothe where we cannot save; and even if the patients are not directly benefitted, their indirect gain will be great if they be preserved from the snares of ignorant imposters.

There are some special reasons why, of all the different classes of empirics, the "cancer curers" should attract a large share of public attention. Cancer has been too generally treated and regarded by medical men as incurable, and the frequency of the return of the disease after the removal of a cancerous tumor by the knife is confessed by all honest men. Then there is the natural fear of the knife; and, on the other hand, the bold assertions and confident promises of the cancer curer.—These persons all tell the same story. They exaggerate the pain and danger attending excision; they endeavor to persuade the public, and even their patients, that the use of their caustics is not attended with much pain, and with no loss of blood. They call innocent tumors cancer. They conceal unsuccessful cases; so do those who were ashamed of having been imposed on; and medical men will not incur the charge of jealous rivalry by making them known. Lastly, they assert that, while relapse is the rule after excision, it is the exception after the removal by their caustics; and they have a theory, which looks plausible enough to the public, explaining why this should be: they say they remove the roots, which the knife does not.

This notion of the roots of cancers, leads me to say something about Plunket and Guy, cancer curers of the past century, who adopted it, just as it has been adopted by two American physicians, Dr, Pattison and Dr. Fell, who have treated cancers by secret remedies in London for some years past. The notion is, that their applications not only destroy the tumor itself, but penetrate, by a sort of intelligent power, or elective affinity, in certain directions, corresponding exactly with these supposed roots of the cancer—eating away or drawing out those roots, without affecting the sound flesh into which they are ingrafted. On removing such tumors they show filaments of hardened cellular tissues, or portions of subjacent muscle, keeping up the connection; and on the tumors they preserve in bottles, they show similar prolongations, or shreds, hanging into the spirit in which the tumors are preserved. These are, in all probability, merely portions of the surrounding tissues which have been destroyed by the action of the caustic. Possibly these supposed roots may have given rise to the tern "cancer"—the crab holding firmly with its claws the prey it had grasped. However this may be, you can see at once how likely such reasoning is to affect the imagination of patients.

Plunket practiced as a cancer curer in London, in the ealry part of last century. He is said to have known little or nothing of surgery in general, and to have practiced from the traditionary directions of his namesake, formerly an empiric in Ireland, who left the receipt for his medicine, with direction for its use, to Steven's hospital. Guy, who was a member of the "Corporation of surgeons," purchased the secret of Plunket about 1754, and in his account of the medicine, says it had been known by the name of "Plunket's poultise," and had been used by Plunket and his ancestors for more than a century. A controversy took place between Guy and Gataker, and in the Lloyd's Evening Post, March 5th, 1760, old Plunket gives his own receipt, as follows: Crow's foot, which grows in low ground, one handful, dog fennel, three sprigs, well poundered; crude brimstone, three middling thimbles full; white arcenic, the same quantity—all incorporated well in a morter, then made into small balls, the size of nutmegs, and dried in the sun.

Sir Charles Blicke, with whom Abernethy served his apprenticeship used Plunket's caustic very much in the treatment of cancerous sores' and his pupils used to be employed in gathering ranunculus and dog fennel, and making them into the paste.

It is curious to remark how imitative even great discoverers may be.—The escharatic effects of arsenic had been known to the Greek and Roman physicians—they had not been forgotten in the middle ages. The mineral had been used for centuries in the removal of cancerous diseases.—Plunket adds some crow's foot and dogfennel to it, and becomes a great cancer curer in London. The chloride of zinc is proved to be an excellent caustic, by Hancke, Canquoin, Alexander Ure, and others. They even use it to remove malignant groths. Dr. Fell adds some sanguinaria conadensis to it, and four gentlemen of the very highest character and professional position, expressing no disapproval of the use of a secret remedy, and without *trial* of the unaided powers of the vegetable, publish a certificate on Dr. Fell's "mode of treatment," complimenting it as "ingenious, safe, and easy of application."

It was Guy's caustic, or rather the Plunket's paste, that killed Lord Bolingbroke, and many others were poisoned by the local use of arsenic; yet this did not prevent Lord Arundel from buying the receipt of the wife of a blacksmith, so ignorant that she could not sign her name, but a noted cancer curer, named Elizabeth Fellow. This was long known as Lord Arundel's cancer cure. It was an arcenical powder, and a wash of corrosive sublimate, and no doubt killed a number of poor women.—However, like Plunket's paste, a great many cancerous and other tumors were removed entire by it; and Mr. Justamond, who was surgeon to the Westminister hospital some seventy or eighty years ago, tried them both very extensively, arriving at the conclusion that the advantage gained did not compensate for the risk incurred. * * * *

Landolfi, a Neapolitan physician, may be looked upon as the prince of the cancer curers. He has been decorated with orders of knighthood by sovereign princes, has been alternately flattered and abused, and has made an immense fortune. He made no secret of his plan. "Landolfi's paste," as his caustic was called, was composed of equal parts of the chlorides of zinc, brimstone, gold and antimony, made into a paste with flour or liquorice powder. Sometimes he used the cloride of bromine alone, using it both externally and internally; and when the slough had been formed, he used lettuce poultices till it separated. There can be no doubt that Landolfi removed an immense number of cancerous tumors by his paste in Italy, Germany and France, and that healthy granulations sprung up, and firm cicatrices very often resulted. He used to assert that out of four thousand caces of cancer he had treated, the disease had not recurred in three thousand. This was what he *said*. He never offered any thing like *proof* of the truth of this statement; and when his caustic was tried in the hospitals of Vienna and Paris the conclusions arrived at were that it was decidedly inferior to the chloride of zinc. Landolfi went himself to Paris, and a number of patients were treated by him in the Salpetriere, under the inspection of a committee of hospital surgeons. Their report was published, and my colleague, Dr. Deville, has just favored me with a copy. The conclusions are, that the chloride of bromine, which is the only peculiarity in Landolfi's treatment, is quite useless as an internal remedy; and that locally it only acts as a blister, raising the epidermis, and exposing the denuded part to the action of the chlorides of zinc and antimony; acting, you observe, just as the ranunculus did in Plunket's paste, the nitrate of silver as used by Justamond, or like any common blister. The committee reported that the pain produced by this caustic was excessive, and that it did not secure the patients from the danger of erysipelas or hemorrhage. Landolfi does not appear to have been more successful in Germany than in France. In November 1853, he was called to the reigning duchess of Anhalt-Cothen, to remove a cancer of the breast. In January 1854, Dr. Brunn, a member of the superior medical council of the duchy, published a pamphlet on Landolfi and his method, in which he announced his success as complete; yet on the 13th of July 1855, the duchess died of a return of the cancer of which Landolfi had cured her. Other cases treated at Cothen and Munich died or relapsed. He treated Dr. Seyfert at Dresden, and he died. He treated a prince of Prussia, and was decorated with the order of the Red Eagle; but here again *cure* meant *cicatrization;* for I have been assured that the disease was cancroid of the face, and that it has returned. Dr. Valentini of Berlin tried the method in 43 cases, and published an article in July 1854 in its favor, but in July 1855, only one year later, he wrote to say that it had entirely disappointed him. So at Vienna, in October 1854, Dr. Weinberger published reports of 33 cases treated before him by Landolfi. One of the cases reported as cured relapsed while Dr. Weinberger was correcting his proof; and ten months later he wrote, that in cases of medullary cancer the disease "always returned, even before the cicatrization of the wound," and that the internal use of the chloride ef bromine had no influence whatever in preventing relapse. Landolfi by ministerial authority *selected* six cases himself at the Vienna hospital, and treated them under the observation of a committee, yet he only cured one, and that was an innocent tumor, a partial hypertrophy of the mamma, for which he destroyed the whole breast quite unnecessarily, and produced a large unsound cicatrix. He wrote to the French commission to say that the effects of the application of his caustic in France were in all respects similar to those he had obtained in Germany and Italy; and so we fine them. Of nine caces of cancer of the breast treated by Landolfi himself at the Salpetriere, two died; in four, the disease was aggravated; and in the three in which cicatrization took place, the disease reappeared. Nor one of the nine was cured. He treated three cases of cancroid, and cured one. In a second, the disease reappeared after cicatrization, and in the third it was much aggravated. So much for the three thousand cures of four thousand cases. Well may the French committee add that Landolfi's method "adds another to the illusions that so abound in the history of cancer."

Dr. Pattison, as you may be aware, some three, four, or five years ago, occupied much the same position in London that Dr. Fell does now.—Both are physicians with American diplomas, who have professed to cure cancer by secret remedies, who have treated a great many patients, and have published accounts of their treatment. The difference between them is, that Dr. Fell has at length made known the composition of the remedies he employs, while Dr. Pattison has not; although it is pretty generally believed, and not without ground, that the essential part of his preparations was the dried sulphate of zinc, which Dr. Simpson showed in the Medical Times and Gazette a few months ago was a most useful caustic. Dr. Pattison has not been heard of so much since the arrival of Dr. Fell. Indeed the disappearance of one and the advent of the other supposed not to have been altogether without concert. Where Dr. Pattison may be now, I cannot say, but his publications remain; and I can tell you something about some of the cases he has treated. A report of one of these used to appear in the form of a declaration sworn before the provost of Glasgow, that the patient was cured by Dr. Pattison, after having been regarded by Mr. Syme of Edinburgh as hopeless. Mr. Syme informs me that the patient was a small farmer, who had a sore at the corner of his nose. Mr. Syme applied the chloride of zinc to it, but the man went to Dr. Pattison, and so far from having been cured by him "died in great misery after several journeys to London." Mr. Syme informs me that a case of cancer of the breast which had returned after operation, and which Dr. Pattison boasted he had cured, is not cured, but that the lady is dying; and that another lady, upon whom Mr. Syme declined to operate for cancer of the tongue, died under Dr. Pattison's care. These are cases which the public never hear of, but which really ought to be made known. In 1855 a book appeared, entitled "Cancer; its true Nature, Treatment and Cure. Illustrated by Cases. By John Pattison, M. D. 31 Dower Grosvenor street." Most of these cases are given so indefinitely, as "Mrs. H. from Essex," "Mrs. J. aged 54," "Miss———, age 27," "Mrs. A. of Hammersmith," "D. C. from Scotland," and so on, that it is impossible to find out how far the cures related are correct; but there is a clue to some of the cases, particularly to those treated at Glasgow; and I wrote to Dr. Macleod, a most able surgeon of Glasgow, to ask him to make enquiries about them.*

I am sure, gentlemen, I need not trouble you more with Dr. Pattison's cures. I have made enquiries myself about others, and I have only found *one* in which the cure was permanent, and that was a case of a small superficial sore on the face, possibly malignant, possibly not.

I do not mean to say much about Dr. Fell. His position is somewhat peculiar; for though he used a secret remedy, he was very open in exhibiting its effects to medical men, and he has lately made known its composition, in compliance with an agreement entered into with the surgeons of the Middlesex hospital, in a book he has recently published. In his preface he talks of the "gratitude of a multitude of cure patients;" but, as the treatment of the earliest case he has recorded as treated in England was commenced July 1855, and the lady died with pulmonary complication in the following April, and the other cases are of much later date, it is obviously absurd to talk of *curers*, when only a few months have elapsed after cicatrization. I know of cases in which the disease has returned after removal by Dr. Fell himself, and that in a much shorter period than two years; and, looking upon the essential part of his treatment to be the local use of chloride of zinc, it appears to be most unlikely that the results will differ from those obtained by Canquoin and others by the use of the same caustic. The report of the surgeons of the Middlesex hospital was drawn up within two months after the treatment was commenced there. I may tell you that Dr. Fell has not published all his formulæ in his book. At the Middlesex hospital he uses an ointment containing snuff and acetate of copper, and another mode by boiling stramonium leaves in lard; but the chloride of zinc is the caustic by which the tumor is destroyed; the sanguinaria and cochineal added to it are probably of nearly equal efficacy, and the after-dressings of comparatively little importance.

All this leads to the very important question of the relative advantages of the knife and caustics in the treatment of cancer. The advocates of caustics say that when the morbid growth is removed by the knife, if it be really cancer, there is almost certain to be a recurrence of the disease within two years, either in the cicatrix or in some other part of the body; that the disease makes more rapid progress than when the patient is left alone, and therefore that he is in a worse position than before. They say,

---

*Of these cases, only two were cured—one a superficial ulcer on the face; the other, a varicose ulcer of the leg.—*Ed.*

further, that it is often impossible to remove every particle of disease tissue with the knife; and that the operation itself is sometimes fatal, either immediately from shock, or indirectly from pyæmia or exhaustion.

On the other hand, the opponents of caustics say, that no caustic can do more than the knife towards removing the cancerous diathesis. They admit that the morbid local growth can be removed, but they assert that the patient is not in a better position, frequently in a worse position, than if the knife had been used. Under the influence of chloroform, the tumor may be removed in a few moments without suffering to the patient, and every portion of diseased tissue can be removed if proper care be taken; while the action of caustic is very slow, often excessively painful, and is sometimes apt to extend beyond the diseased to the healthy surrounding structures. They add, that during the operation of the caustic the patient is not free from the danger of hæmorrhage, erysipelas, or pyæmia, and that the general health is very apt to suffer from the long continued pain and local irritation.

Now I am disposed to look upon all these arguments as quite secondary to the great question, which mode of removing the tumor is most likely to be followed by relapse. The advantages and disadvantages of the two methods are pretty equally balanced in other respects; and I apprehend we shall find them, even in this respect, also pretty equal. Statistics collected with a good deal of care show that about eight cases out of every ten operated on by the knife return in two years. Dr. Fell says that only three out of ten treated by him returned within the same period. One good effect of the trial given to his treatment at the Middlesex hospital will be that the truth of this statement will be tested. At present you must take it for what it is worth. Landolfi said something of the same kind, yet I have given you the results he obtained at the Salpetriere.—Pattison harped on the same string, and I have shown you how far he succeeded in preventing relapse. As to Plunket and Guy, and those who used arcenic, I need only remind you of the fact that their remedies have fallen into disuse, although they were employed long enough to establish their reputation all over the world, had they really possessed the powers attributed to them of curing cancer.—*Va. Med. Journal.*

